# Mapping the Risk for West Nile Virus Transmission, Africa

**DOI:** 10.3201/eid2804.211103

**Published:** 2022-04

**Authors:** José-María García-Carrasco, Antonio-Román Muñoz, Jesús Olivero, Marina Segura, Raimundo Real

**Affiliations:** University of Malaga, Malaga, Spain (J.-M. García-Carrasco, A.-R. Muñoz, J. Olivero, R. Real);; Ministry of Health, Consumption, and Social Welfare, Malaga (M. Segura)

**Keywords:** West Nile virus, arbovirus, epidemic, epizootic, infectious disease, pathogeography, zoonoses, viruses, Africa, vector-borne infections

## Abstract

West Nile virus (WNV) is an emergent arthropodborne virus that is transmitted from bird to bird by mosquitoes. Spillover events occur when infected mosquitoes bite mammals. We created a geopositioned database of WNV presence in Africa and considered reports of the virus in all animal components: reservoirs, vectors, and nonhuman dead-end hosts. We built various biogeographic models to determine which drivers explain the distribution of WNV throughout Africa. Wetlands of international importance for birds accounted for the detection of WNV in all animal components, whereas human-related drivers played a key role in the epizootic cases. We combined these models to obtain an integrative and large-scale perspective of the areas at risk for WNV spillover. Understanding which areas pose the highest risk would enable us to address the management of this spreading disease and to comprehend the translocation of WNV outside Africa through avian migration routes.

West Nile virus (WNV) is one of the most widespread of the arboviruses because of the translocation of the virus by migratory birds (*1*–*3*). Since its initial detection in Uganda in 1937 (*4*), WNV has spread throughout much of Africa (*5*,*6*), Europe (*7*), West Asia (*8*), Oceania (*9*), and the Americas (*10*,*11*). The enzootic cycle is maintained between birds (the reservoirs) and mainly mosquitoes (the vectors), whereas humans are accidental dead-end hosts (Figure 1). Other mammals such as horses, dogs, camels, and goats are also accidental dead-end hosts for WNV (*12*). The role of animal monitoring in the surveillance of WNV outbreaks is critical because detecting the virus in animals can help to anticipate its transmission to humans. Moreover, domestic animals such as horses (*13*) and poultry (*14*) have been used as sentinels for human cases. Furthermore, wild birds such as crows have been used to define the geographic and temporal limits of WNV in North America (*15*).

**Figure 1:**
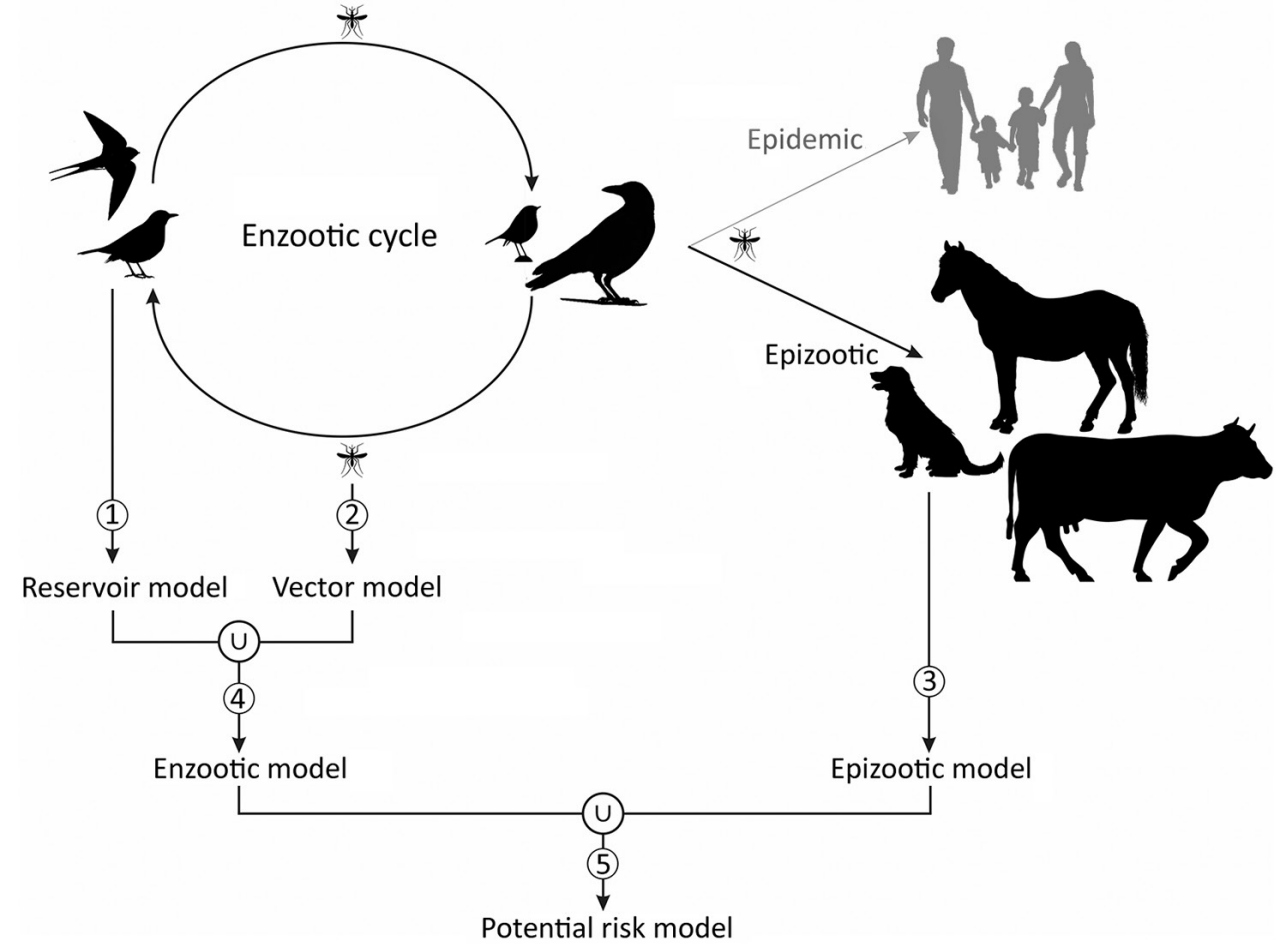
Lifecycle of West Nile virus and schematic elaboration of different models (numbered 1–5) for each component of the cycle of models for Africa. Model 1 (reservoir model) identifies favorable areas for the virus presence in reservoir animals. Model 2 (vector model) identifies favorable areas for the virus presence in vector animals. Model 3 (epizootic model) identifies favorable areas for the virus presence in dead-end hosts. Model 4 (enzootic model) is a fuzzy union of the reservoir and vector models, identifying areas favorable for the virus presence in the reservoir or vector animals. Model 5 (potential risk model) is a fuzzy union of the enzootic and the epizootic models, identifying areas with potential for virus spillovers.

Because WNV does not produce specific clinical symptoms, WNV infection can be mistaken for other infectious diseases and toxins (*16*). WNV outbreaks can easily be attributed to other arbovirus diseases that are more common and result in greater human illness in an area. For this reason, any evidence regarding the presence of WNV in an area is important to ensure monitoring of the risk for humans contracting the disease caused by WNV. Thus, all WNV reports should serve as suitable input data for pathogeographic analyses (*17*) aimed at mapping the areas at risk for WNV transmission to humans.

We conducted a bibliographic review of the detection of WNV in animals in Africa. Next, we applied biogeographic methods to create empirical models on the basis of the virus lifecycle to identify zones that are environmentally favorable for the circulation of WNV in Africa. Moreover, the models were used to ascertain the potential risk for transmission of WNV to animals (epizootic processes) and humans (epidemic processes), even in regions where WNV has not yet been detected.

## Materials and Methods

### Data Sources and Search Strategy

**Figure 2:**
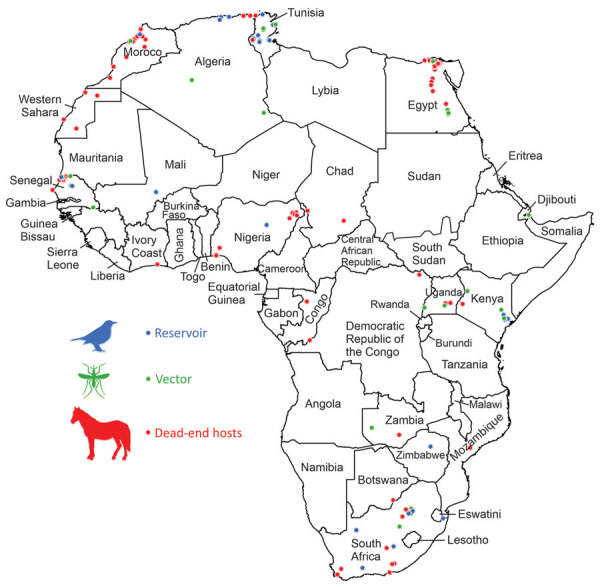
Geoposition of West Nile virus reports in reservoirs, vectors, and nonhuman mammal dead-end hosts, Africa.

We performed a literature search in the GIDEON database (*18*) for 48 countries and territories of Africa (Figure 2), using “West Nile fever” and country names as keywords. For countries that had name changes since 1937, when WNV was first described, we also searched for the ancient names or names that they were otherwise known by; for example, Equatorial Guinea (formerly Spanish Guinea), Saharawi Arab Democratic Republic (Western Sahara), and Côte d’Ivoire (Ivory Coast). We excluded the island countries and territories of Africa from this analysis because WNV probably would be enzootic and independent of the annual movements of migratory birds. The size and isolation of some of these island countries and territories would deserve an independent approach to study WNV (*19*). We complemented the reports obtained from GIDEON with articles acquired through an electronic literature search of the Web of Science (https://clarivate.com/webofsciencegroup/solutions/web-of-science), Scopus (https://www.scopus.com), and Google Scholar (https://scholar.google.com) for all countries in Africa, for which we used different combinations of the following keywords: “West Nile virus,” “WNV,” “West Nile Fever,” “WNF,” and the name of each country. The reports and articles obtained provided a selection of geopositioned reports that described the presence of WNV in vectors (principally mosquitoes), reservoirs (i.e., birds) and dead-end hosts (i.e., horses, dogs, and other mammals, excluding humans). To obtain a robust high-resolution database, we took into account occurrences of WNV only when the reports referred to specific villages, towns, or cities. We used the names of the localities and the contextual information provided in the information sources to determine the latitudinal and longitudinal coordinates, using Google Maps (https://www.google.es/maps), Google Earth (https://www.google.com/intl/es/earth), Geonames (http://www.geonames.org), and Google Search (https://www.google.com).

### Analysis

To reduce the excessive weight of the oversampled areas in the analysis and, thus, autocorrelation caused by sampling bias, we projected the occurrences of WNV onto a grid of equal-sized hexagonal units of 7,742 km^2^. We created a total of 3,970 hexagons by using Discrete Global Grids for R (*20*). If a report of WNV was located within a hexagon, we considered this report to represent a single presence, regardless of the number of records included, whereas we considered the hexagons that did not contain a report of WNV to represent absences. In this way, the hexagons were operational geographic units (OGUs).

We used a set of environmental variables to identify the areas in Africa that were favorable for the presence of the WNV (Appendix 1 Table 1). We classified variables as anthropic (human-related) (e.g., infrastructure or agriculture) or nonanthropic (e.g., climate and ecosystem). The ecosystem variables comprised land cover and Ramsar sites (i.e., wetlands of international importance for birds, as determined by a 1971 treaty signed in Ramsar, Iran). These variables could influence the enzootic (reservoirs and vectors) and epizootic (animal dead-end hosts) components of the cycle of WNV, or they could be correlated with drivers of the presence of these components. For each OGU, we calculated an average value for every explanatory variable through the Zonal Statistic as Table tool from ArcGIS Desktop 10.7 software (ESRI, https://www.esri.com). We used biogeographic modeling based on fuzzy logic and machine learning algorithms to separately analyze the environmental characteristics of WNV in every component of its cycle (Figure 1).

We developed 3 biogeographic models. First was the reservoir model (Figure 1), based on the presence of WNV in reservoir animals, which we intended to detect areas that were favorable to birds becoming infected by WNV. Second was the vector model (Figure 1), which was based on the presence of WNV in vector animals and identified areas favorable to WNV detection in mosquitoes. Third was the epizootic model (Figure 1), based on the presence of WNV in nonhuman mammals, which aimed to detect the areas in which environmental conditions could lead to WNV spillover. To address a comprehensive biogeographic approach to WNV in Africa in the context of reservoir–vector relationships, we identified the areas that are favorable for the presence of WNV in reservoirs or vectors. Accordingly, we joined the reservoir and vector models into a single enzootic model (Figure 1) by calculating their fuzzy union (i.e., the maximum favorability value for any of them [F-reservoir model ∪ F-vector model]). Finally, we merged the enzootic and the epizootic models into a WNV potential risk model (Figure 1), which represented the fuzzy set of areas where the environment is favorable for the presence of WNV in reservoirs, vectors, or dead-end hosts. To this end, we performed a fuzzy union of their favorability values (i.e., F-enzootic model ∪ F-epizootic model). Working separately with 3 different models (1 for each component of the WNV cycle), instead of creating a single model based on the detection of WNV in any component of the cycle, enabled us to investigate whether the detection of WNV in the various components could be explained by different drivers. Although the presence of WNV in vectors and dead-end hosts indirectly indicates its presence in reservoirs, this presence may occur at various intensities given the intrinsic characteristics of each of the components of the virus cycle.

We produced each model by using several steps. To control the multicollinearity among the environmental variables, we calculated pairwise Spearman correlation coefficients between all variables. If 2 variables belonging to the same subtype of variables (Appendix 1 Table 1) showed a correlation >0.8, we deleted the least explanatory variable. Considering only the remaining variables, we addressed a false discovery rate control to limit the increase in the type I error caused by the number of variables analyzed (*21*). Hence, we arranged the variables in decreasing order according to their relevance in explaining the presence of WNV. We assessed this relevance according to Rao score tests (*22*). A variable was used in subsequent steps only if its score-test probability was lower than i*q/V (where i is the position of the variable in the referred order, q = 0.05 is the false discovery rate, and V is the total number of remaining variables).

We used all variables that advanced through the previous filters in a multivariate stepwise logistic regression, a commonly used machine learning algorithm (*23*), that began with a null model that had no explanatory variables included. We then added a variable at each step if the resulting new regression was significantly improved by the new variable. The result of the multivariate logistic regression was a probability value of WNV being present in each OGU according to the environmental characteristics of the OGU. We transformed the probability values of each OGU into favorability values by using the favorability function (*24*) (Appendix 1). A more detailed discussion of the procedure has been published previously (*25*).

We evaluated the discrimination and classification capacities of each model. We assessed the model discrimination capacity by using the area under the receiver operating characteristic curve (*26*). We estimated the classification power by using the value *F* = 0.5 as a classification threshold through sensitivity, specificity, Cohen κ, the correct classification rate (*27*), and the overprediction and underprediction rates (*28*). Finally, we compared the performance of the potential risk model to that of an alternative risk model that was based on the use of the entire set of WNV occurrences (i.e., those occurrences reported in birds, mosquitoes, and mammals) as the dependent variable. Considering that WNV outbreaks in Africa are underestimated because of the generic symptomatology (*16*,*29*), an effective model should demonstrate high sensitivity and a low underprediction rate to detect potential risk areas. We projected the distribution of WNV by using the geographic information system ArcGIS Desktop 10.7 and performed logistic regressions by using SPSS Statistics 26 (https://www.ibm.com).

## Results

### Database

Among 328 articles identified during the literature search, we included 71 in the analysis. We excluded the remainder for any of the following reasons: the survey for WNV was negative for this specific virus, the survey for WNV was positive for the virus but the research was conducted in an entire region, or the survey was conducted to a country level without identifying a specific place.

We collected 189 geopositioned localities where the WNV was present: 33 locations where WNV occurred in reservoirs, 48 locations where WNV was detected in vectors, and 108 locations where WNV occurred in dead-end hosts. These localities were included in 83 of the 3,970 OGUs, and they were distributed across 20 countries in Africa. The presence of WNV in reservoirs (Figure 2; Appendix 2) involved 52 species and 10 orders of birds. The presence of WNV in vectors (Figure 2; Appendix 2) involved 23 mosquito species and 1 tick species (*Argas reflexus hermanni*). In certain cases, only the genus of the mosquito pool was identified: *Culex* and *Aedes*. Finally, the presence of WNV in dead-end hosts (Figure 2; Appendix 2) mostly involved equids and dogs, although WNV was also detected in bats, buffaloes, camels, monkeys, and elephants.

### Biogeographic models

**Figure 3:**
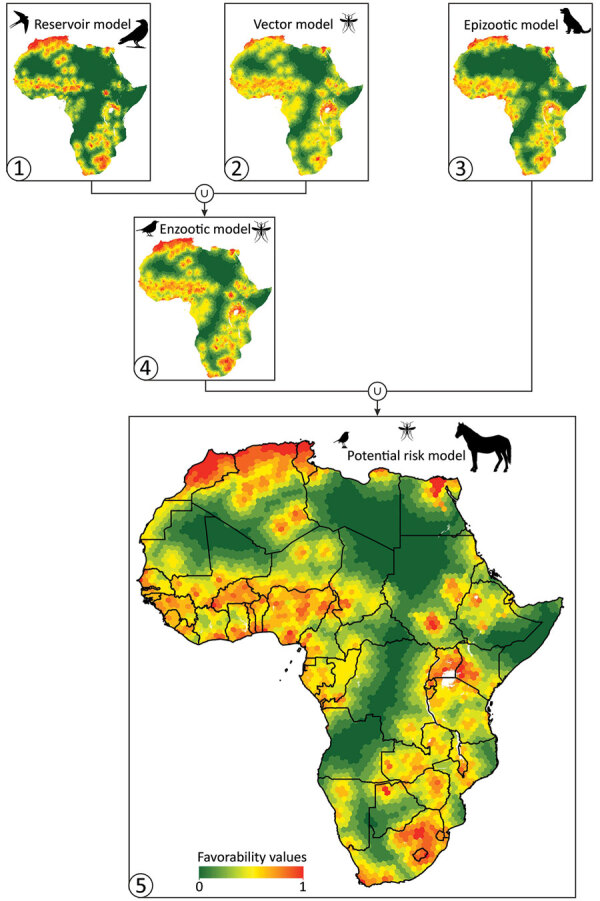
Cartographic representation of the biogeographic models (numbered 1–5) based on the different West Nile virus lifecycle components for Africa. Model 1 (reservoir model) indicates environmental favorability for the presence of the virus in birds. Model 2 (vector model) indicates environmental favorability for the presence of the virus in vectors. Model 3 (epizootic model) indicates environmental favorability for the presence of the virus in nonhuman mammals. Model 4 (enzootic model) indicates environmental favorability for the presence of the virus in >1 component of the enzootic virus cycle. Model 5 (potential risk model) indicates environmental favorability for potential spillover of the virus.

The most favorable areas on the continent for WNV-infected birds were located in Northern Africa (specifically Morocco, northern Algeria, Tunisia, and the Nile Delta), West Africa, and southern Africa (Figure 3). Reservoir zones were characterized climatically by high minimum temperatures (B = −1.16 × 10^−2^), ecosystemically by being close to Ramsar sites (B = −0.96), and having vegetation on regularly flooded soil (B = 5.39) and anthropically by the presence of croplands (B = 2.78) and high densities of poultry (B = 1.00 × 10^−5^) (Table).

**Table:** Predictor variables included in reservoir, vector, and dead-end host West Nile fever models for Africa*

Variable	Reservoir			Vector			Dead-end host	
	B	Wald		B	Wald		B	Wald
Climatic								
Minimum temperature of the coldest month	(−) 1.16 × 10^−2^	9.65						
Ecosystemic								
Distance to Ramsar sites	(−) 0.96	8.52		(−) 0.54	6.49		(−) 0.68	13.59
Vegetation on flooded soil	(+) 5.39	4.51						
Human								
Cropland and vegetation	(+) 2.78	6.27						
% Of irrigation areas							(+) 0.04	5.06
Chicken density	(+) 1.00 × 10^−5^	13.71		(+) 1.60 × 10^−5^	27.15		(+) 1.20 × 10^−5^	13.49
Cattle density				(+) 1.78 × 10^−5^	4.32			
Population density							(+) 1.10 × 10^−3^	7.11
Distance to railway							(−) 3.00 × 10^−3^	4.60

*Signs in parentheses indicate positive/negative relationships between favorability and variables. B is the coefficient multiplying the variable values in the logit of the multivariate logistic regression. The Wald parameter quantifies the relevance of every variable in the model. Variable abbreviations are given in Appendix 1 Table 1.

The favorable areas for vectors to become infected with the WNV were not unlike those shown by the reservoir model. Nevertheless, the areas with high environmental favorability (*F*>0.8) were less extensive, whereas the intermediate-favorability zones (*F* = 0.2–0.8) were wider, including the areas around Lake Victoria where WNV was isolated for the first time (Figure 3). Favorable areas in the vector model were characterized by their ecosystemic and anthropic conditions: the closeness to Ramsar sites (B = −0.54), and the poultry (B = 1.60 × 10^−5^) and cattle density (B = 1.78 × 10^−5^) (Table). The enzootic model, derived from a combination of the reservoir and vector models, included all the areas that were environmentally favorable for WNV to be present in birds or arthropods (Figure 3).

According to the epizootic model, the areas most prone to experiencing epizootic outbreaks were geographically similar to those that were highlighted as favorable by the reservoir and vector models (Figure 3). However, the subtle geographic differences between the epizootic model and the others led to the inclusion of a different pool of variables. Specifically, the most important predictors were anthropic variables, such as a high-density human population (B = 1.10 × 10^−3^), the proximity to railway tracks (B = −3.00 × 10^−6^), a high density of poultry (B = 1.20 × 10^−5^), and a high percentage of irrigated crops (B = 0.04). The short distance to the Ramsar sites (B = −0.68) also explained the distribution of the epizootic cases that were reported (Table).

The model that defined the areas with a potential risk for WNV transmission to dead-end hosts, including humans, comprised all areas that were favorable for the presence of WNV in >1 of the components of the cycle of WNV (Figures 1, 3). The risk extended throughout most of the continent, except the desert areas in the Horn of Africa (Sahara and Kalahari) that are far from oases and a strip that runs north to south along central Africa, between Sudan and Angola (Figure 3).

The potential risk model provided a more informative cartographic output compared with the single model that comprised all WNV detections. In addition, the potential risk model demonstrated an improved sensitivity (0.87) and underprediction rate (0.0042) compared with the alternative model (sensitivity 0.84, underprediction rate 0.0045) (Appendix 1 Table 2).

## Discussion

Our review sought to obtain a broad perspective regarding the geographic distribution of WNV throughout the continent of Africa. Previous studies addressed the distribution of WNV in Africa at a country level (*30*–*32*) or considered subcontinental contexts such as the Eastern Mediterranean area (*8*). Nevertheless, our study analyzed the geography of the potential health risks, derived from the distribution of WNV at a fine (<8.000 m^2^) spatial resolution throughout the entire continent, which elucidates the international risk patterns on this continent. Moreover, our study is useful for understanding the patterns of virus expansion in the continent of Africa and the seasonality patterns that occur in Europe (*25*).

The geopositioning of the locations in Africa where WNV has been detected (at various stages of its lifecycle) has enabled us to assume different considerations to develop a risk model of the WNV for the entire continent. The presence of WNV in mosquitoes and birds enabled us to develop an enzootic model. We identified the environmental drivers that favor the enzootic circulation of WNV and the most favorable for its circulation. However, WNV can also experience spillover events in mammals (epizootic cycle). Knowing the environmental characteristics that promote these spillovers enabled us to identify where the most susceptible areas to virus transmission are that exceed the enzootic cycle. In addition, by considering the favorable areas for virus transmission in the enzootic and epizootic cycles, we created a potential risk map that highlights the areas where WNV is most likely present (in >1 components of the WNV cycle) and can ultimately lead to spillover to dead-end mammal hosts, including humans.

The variables involved in the distribution of WNV in Africa are associated with the climate, ecosystems, and human activity (Table). However, the proximity of the Ramsar sites contributed to an explanation of the presence of WNV in each of the components of the virus cycle (i.e., reservoirs, vectors, and mammals). In Tunisia, the proximity to the Ramsar sites was important for explaining the occurrence of WNV in horses (*30*) and humans (*31*). Given the protection and conservation status of the Ramsar sites, they offer an ideal habitat for sedentary and migratory birds (which can carry WNV) (*33*) and for mosquitoes (*34*). Therefore, we are not surprised that their proximity partially explains the detection of WNV in birds, mosquitoes, and mammals. Except for the proximity to the Ramsar sites, the remaining explanatory variables included in the epizootic model were associated with human activity (Table). Most WNV detections outside the enzootic cycle have been observed in domestic animals, such as horses and dogs. Thus, the favorable areas for the presence of WNV in the epizootic cycle may also reveal the risk for spillover to humans and other mammals.

Because cases of WNV are generally underestimated, we aimed to develop a model with high sensitivity and a low underprediction rate so that potential risk areas would not be ignored. Our potential risk model that resulted from the fuzzy union of the enzootic and epizootic models had a higher sensitivity and lower underprediction rate than the alternative model that considered all the occurrences of WNV presences. This approach demonstrated the convenience of a macro-ecologic perspective that integrates all components of the lifecycle of a pathogen to obtain a comprehensive understanding of risks associated with zoonotic diseases.

In the middle of the Sahara Desert, the favorable zones for WNV (Figure 3) correspond to the National Parks of Ahaggar and Tassili n’Ajjer in Algeria and the oases of Kawar and l’Aïr in Niger, where inhospitable conditions are less extreme than in the rest of the desert. Moreover, in these areas, closer contact probably occurs between avian hosts and mosquitoes around the remaining water sources, favoring the enzootic cycle (*35*,*36*).

Applying biogeographic models to zoonotic diseases helps detect areas that pose a risk for disease transmission. However, these models may have certain limitations. The disease reservoirs may have a great dispersal capacity, especially long-distance migratory birds. In our case, we considered the place where the WNV-positive sample was recorded, although the bird could have been infected in other parts of the continent. WNV is a neglected disease; reports on its detection in vectors, reservoirs, and dead-end hosts are limited. The relatively low number of locations in such a large study area may lead to a map that underestimates the potential risk. However, our model highlighted areas with a high risk for WNV in countries where it has not been detected yet, such as Burundi, Lesotho, Eswatini, The Gambia, Guinea-Bissau, Togo, Benin, and Malawi.

The potential risk model could reveal the risk not only to animals but also to humans because it characterizes the environmental conditions in which spillovers occur. Northwestern Morocco is an area where human WNV cases have occurred repeatedly (*37*,*38*) and was highlighted as a high-risk area in our model. The same situation occurs in Tunisia (*29*,*37*) and along the Nile River in Egypt (*39*), particularly in the Nile Delta (*40*). Our model predicted high-risk areas for WNV in the center and the south of Algeria, in isolated areas that correspond to oases. Furthermore, human cases of WNV occurred in Timimoun (in the center) (*34*,*37*) and Djanet and Tamanrasset (in the south) (*34*). In Uganda (*41*,*42*) and South Africa, human cases have also been reported, particularly in Pretoria and Johannesburg (*43*), which were highlighted in our model as the areas with the highest risk.

Recognizing the conditions that favor the onset of WNV would enable us to optimize resources to prevent the disease. For example, the percentage of irrigation areas (Table) is positively correlated with epizootic episodes. Therefore, during the transmission season, resources to address prevention policies should be put in place in agricultural areas that use irrigation systems. Given the role of bird migration in the spread of viruses, including WNV (*2*), maintaining a broad spatial perspective and an improved understanding regarding the contribution of the movements of hosts in the spread of the disease is important. Knowing the favorable areas for the presence of WNV in its continent of origin may be of great help for disease prevention at an international level. This knowledge may aid in managing the disease from an intercontinental perspective. Our model may help provide improved medical advice to persons traveling to the area, including screening for WNV upon return to the traveler’s native country, because no vaccines are available for use in humans (*44*,*45*). 

Areas of North Africa are important stopover sites for migratory birds and are areas of high enzootic risk. Because WNV viremia in birds can last for up to 7 days (*46*,*47*), birds could become infected in these areas and arrive in Europe with a viral load high enough to introduce WNV to Southern and Central Europe. Nowadays, WNV is a priority mosquitoborne pathogen that is spreading in Europe (*3*,*25*,*48*,*49*). Therefore, knowing the favorable zones for WNV in the wintering and breeding areas of migratory birds may lead to an understanding of the evolution of WNV and help to prevent outbreaks in Europe. 

Predicting zoonotic disease outbreaks is one of the ultimate challenges for public health management and the primary goal of preventive medicine (*17*). Therefore, developing WNV risk maps that account for the dynamic biogeography of birds can help prevent the disease or lead to early management responses to reduce the impact of the disease on humans and domestic animals.

Appendix 1 Explanatory variables used in the West Nile virus models, favorability function, and comparative assessment of the classification and discrimination capacities of models for Africa.

Appendix 2 Database and references for database used in the West Nile virus models for Africa.
