# Supplementary material for: Mapping the Risk for West Nile Virus Transmission, Africa
Source: Emerg Infect Dis. 2022 Apr;28(4):777–85. doi: 10.3201/eid2804.211103 (PMC8962882; doi:10.3201/eid2804.211103)
Supplement: Appendix 1 — Explanatory variables used in the West Nile virus models, favorability function, and comparative assessment of the classification and discrimination capacities of models for Africa. [file 21-1103-Techapp-s1.pdf]

Article DOI: https://doi.org/10.3201/eid2804.211103

Mapping the Risk for West Nile Virus Transmission, Africa

Appendix 1

Favorability Function

$$F = \frac{\frac{P}{1-P}}{\frac{n1}{n2} + \frac{P}{1-P}}$$

F being favorability value and P the probability value. n1 is the number of OGUs with reported WNV, and n0 the number of OGUs with no virus reported. The result was a F value (ranging from 0 to 1) in every OGU, representing the degree to which environmental conditions in the OGU favor the occurrence of the virus. For a more detailed procedure, see García-Carrasco et al. (*1*).

Appendix 1 Table 1. Explanatory variables used in the West Nile virus models*

Type	Subtype	Abbreviation	Name
Human	Human	dens_pop	Population density (*2*)
	concentration	distcenpob	Distance to population center (*3*)
	Livestock	chicken	Poultry density (*4*)
		duck	Farmed duck density (*4*)
		horse	Horse density (*4*)
		buffalo	Buffalo density (*4*)
		goat	Goat density (*4*)
		cattle	Cattle density (*4*)
		sheep	Sheep density (*4*)
		pig	Pig density (*4*)
	Infrastructure	dist_road	Distance to roads (*5*)
		dist_rail	Distance to railway tracks (*5*)
	Agriculture	class 11	Irrigated croplands (*6*)
		class 14	Rainfed croplands (*6*)
		Crop_veg 20	Mosaic cropland (>50%)/Vegetation(<50%) (*6*)
		class 30	Mosaic vegetation(>50%)/Cropland (<50%) (*6*)
		irrig	Percentage of areas equipped for irrigation (*7*)
Non-human	Ecosystem	class 40	Broadleaved evergreen and/or semi-deciduous forest (*6*)
		class 50	Closed broadleaved deciuous forest (*6*)
		class 60	Open broadleaved deciuous forest (*6*)
		class 70	Closed needleleaved evergreen forest (*6*)
		class 90	Open needleleaved deciuous or evergreen forest (*6*)
		class 100	Closed to open mixed broadleaved and needleleaved forest (*6*)
		class 110	Mosaic Forest/Shrubland/Grassland (*6*)
		class 120	Mosaic Grassland/Forest/Shrubland (*6*)
		class 130	Shrubland (*6*)
		class 140	Grassland (*6*)
		class 150	Sparse vegetation (*6*)
		class 160	Broadleaved forest regularly flooded (*6*)
		class 170	Broadleaved semi-deciduous and/or evergreen forest regularly flooded (*6*)

Type	Subtype	Abbreviation	Name
		Veg_flood	Vegetation on regularly flooded or waterlogged soil (*6*)
		class 200	Desert (*6*)
		forest_loss	Forest loss (*8*)
		dist_ramsar	Distance to Ramsar sites (*9*)
	Hydrographic	dist_river	Distance to rivers (*10*)
	Topographic	alt	Altitude (*11*)
		slope	Slope (*11*)
	Climatic	Mean_temp	Annual Mean Temperature (*12*)
		Max_temp	Max Temperature of Warmest Month (*12*)
		Min_temp	Min Temperature of Coldest Month (*12*)
		Temp_range	Temperature Annual Range (Bio5-Bio6) (*12*)
		Prec	Annual Precipitation (*12*)
		Prec_season	Precipitation Seasonality (Coefficient of Variation) (*12*)

Appendix 1 Table 2. Comparative assessment of the classification and discrimination capacities of models

Classification	Potential Risk Model	All-presences model
Kappa	0.0626	0.0835
Sensitivity	0.8675	0.8434
Specificity	0.6676	0.7371
CCR	0.6718	0.7393
Underprediction	0.0042	0.0045
Overprediction	0.9472	0.9359
Discrimination		
AUC	0.8612	0.8737

*Favorability value 0.5 used as a cutoff pint for classification purposes.

References

1. García-Carrasco J-M, Muñoz A-R, Olivero J, Segura M, Real R. Predicting the spatio-temporal spread of West Nile virus in Europe. PLoS Negl Trop Dis. 2021;15:e0009022. PubMed https://doi.org/10.1371/journal.pntd.0009022

2. Oak Ridge National Laboratory. LandScan 2008 high resolution global population [cited 2015 Sep 8]. https://landscan.ornl.gov

3. United Nations' Food and Agriculture Organization. Relational World Database II (RWDB2) administrative centres and populated places [cited 2017 Jan 25]. https://data.apps.fao.org/map/catalog/srv/eng/catalog.search#/metadata/93e9ab90-b06d-11db-8922-000d939bc5d8

4. United Nations' Food and Agriculture Organization. Livestock systems [cited 2020 Jun 15]. https://www.fao.org/livestock-systems

5. Harvard University. Vector map level 0. 2002 [cited 2016 Oct 23]. https://worldmap.maps.arcgis.com

6. United Nations' Food and Agriculture Organization. GlobCover land cover maps [cited 20108 Dec 7]. https://www.fao.org/land-water/land/land-governance/land-resources-planning-toolbox/category/details/en/c/1036356

7. United Nations' Food and Agriculture Organization. Global map of irrigation areas (version 4.0.1) [cited 2019 Oct 3]. https://www.fao.org/land-water/databases-and-software

8. University of Maryland. Global Forest Change 2000–2019 [cited 2020 Jul 7]. http://earthenginepartners.appspot.com/science-2013-global-forest/download_v1.7.html

9. Ramsar Sites Information Service. Ramsar sites [cited 2020 May 24]. https://rsis.ramsar.org

10. Center for Global Environmental Research. Global Drainage Basin Database GDBD. Released Version 1.0: May 29, 2007 [cited 2017 Mar 9]. http://www.cger.nies.go.jp/db/gdbd/gdbd_index_e.html

11. US Geological Survey. GTOPO30 (1996) [cited 2015 Feb 11]. https://lta.cr.usgs.gov/GTOPO30

12. CHELSA. Climatologies at high resolution for the Earth land surface areas [cited 2018 Oct 22]. http://chelsa-climate.org
